# Supplementary material for: Root-Associated Microbiomes, Growth and Health of Ornamental Geophytes Treated with Commercial Plant Growth-Promoting Products
Source: Microorganisms. 2021 Aug 23;9(8):1785. doi: 10.3390/microorganisms9081785 (PMC8401597; doi:10.3390/microorganisms9081785)
Supplement: Supplementary file 1 [file microorganisms-09-01785-s001.zip › microorganisms-1334779-supplementary.pdf]

Supplementary Materials:

Root-associated microbiomes, growth and health of ornamental geophytes treated with commercial plant growth-promoting products

Gavriel Friesem [1,2], Noam Reznik [2], Michal Sharon Cohen [2], Nir Carmi [2], Zohar Kerem [3], and Iris Yedidia [2,*]

[1] Department of Agroecology and Plant Health, The Robert H. Smith Faculty of Agriculture, Food and Environment, The Hebrew University of Jerusalem, Rehovot, Israel; gavrielf@volcani.agri.gov.il (G.F.); noamr@volcani.agri.gov.il (N.R.); michal.sharon78@gmail.com (M.S.C.); vhncarmi@volcani.agri.gov.il (N.C.); zohar.kerem@mail.huji.ac.il (Z.K.)

[2] Institute of Plant Sciences, Agricultural Research Organization, Volcani Center, Bet Dagan, Israel;

[3] Department of Biochemistry, Food Science and Nutrition, The Robert H. Smith Faculty of Agriculture, Food and Environment, The Hebrew University of Jerusalem, Rehovot, Israel;

* Correspondence: irisy@volcani.agri.gov.il (I.Y.); Tel.: +972-3-9683387

Received: date; Accepted: date; Published: date

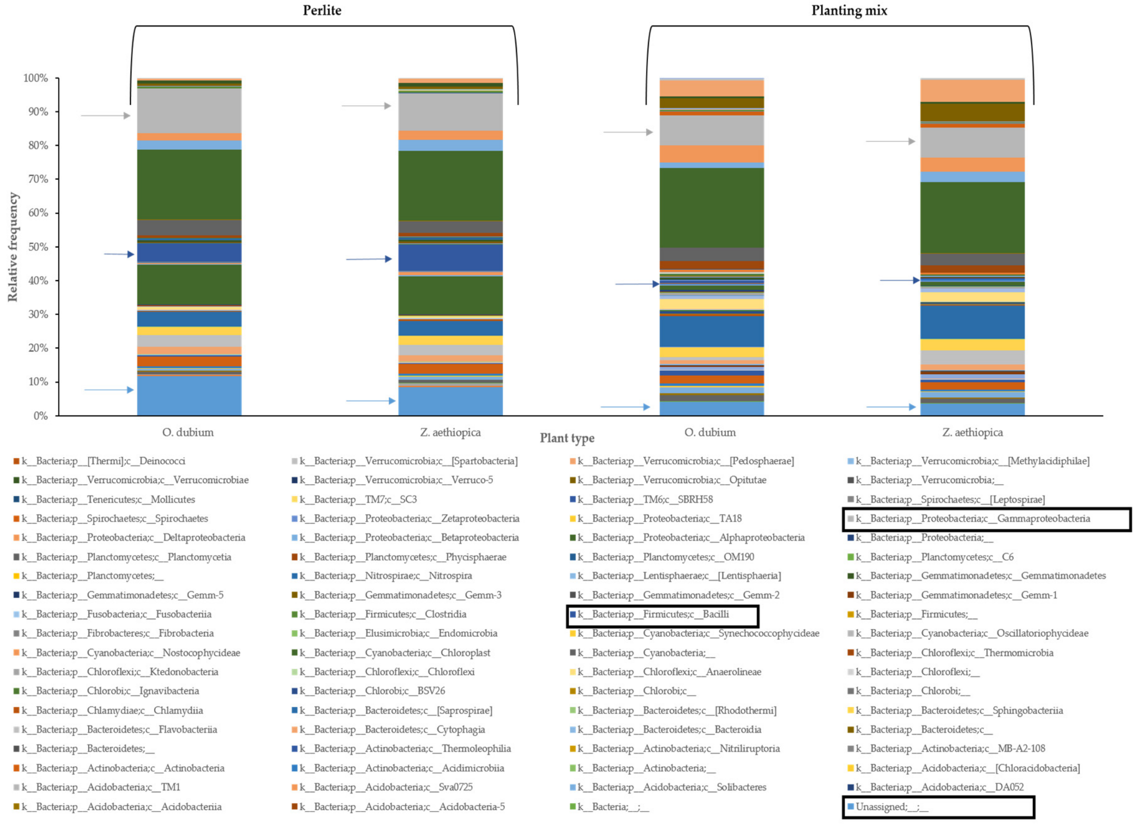

Figure S1. Taxonomic classification (at the class level) of the microorganisms in the root-associated microbiomes of *Ornithogalum dubium* and *Zantedeschia aethiopica*. Data were examined for two growing seasons, two growing media (perlite, planting mix) and the two plant species (*O. dubium*, *Z. aethiopica*). Taxonomic classes are shown in the graphs. The marked classes are *Gammaproteobacteria* (gray square, top

frame) to which *Pectobacterium* belongs, *Bacilli* (deep blue square, middle frame) to which the *Bacillus* spp. found in the Ecosense and Rhizoctol treatments belong, and unassigned sequences (light blue, bottom frame).

A.

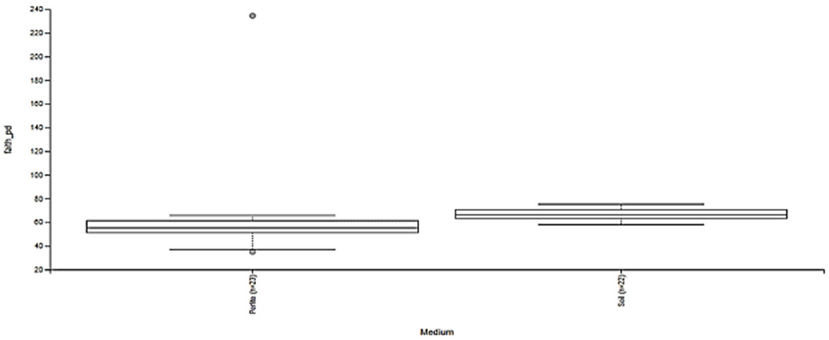

Group 1	Group 2	H	p-value	q-value
Perlite (n=23)	Soil (n=22)	17.836054	0.000024	0.000024

B.

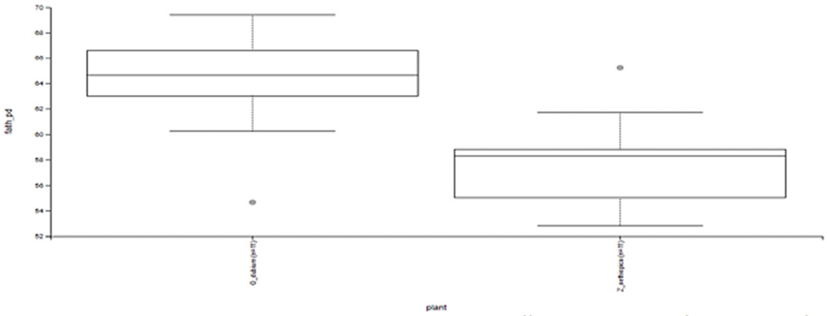

Group 1	Group 2	H	p-value	q-value
O_dubium (n=11)	Z_aethiopica (n=11)	8.926698	0.00281	0.00281

Figure S2. Microbial population dynamics by growing medium and plant species. A Faith PD test was used to compare the bacterial populations in the root-associated environment of two different geophyte species subjected to the different growth-promoting treatments: (**A**) growing medium and (**B**) plant species. Data analysis was performed using variance analysis (PERMANOVA; $p \leq 0.0001$).

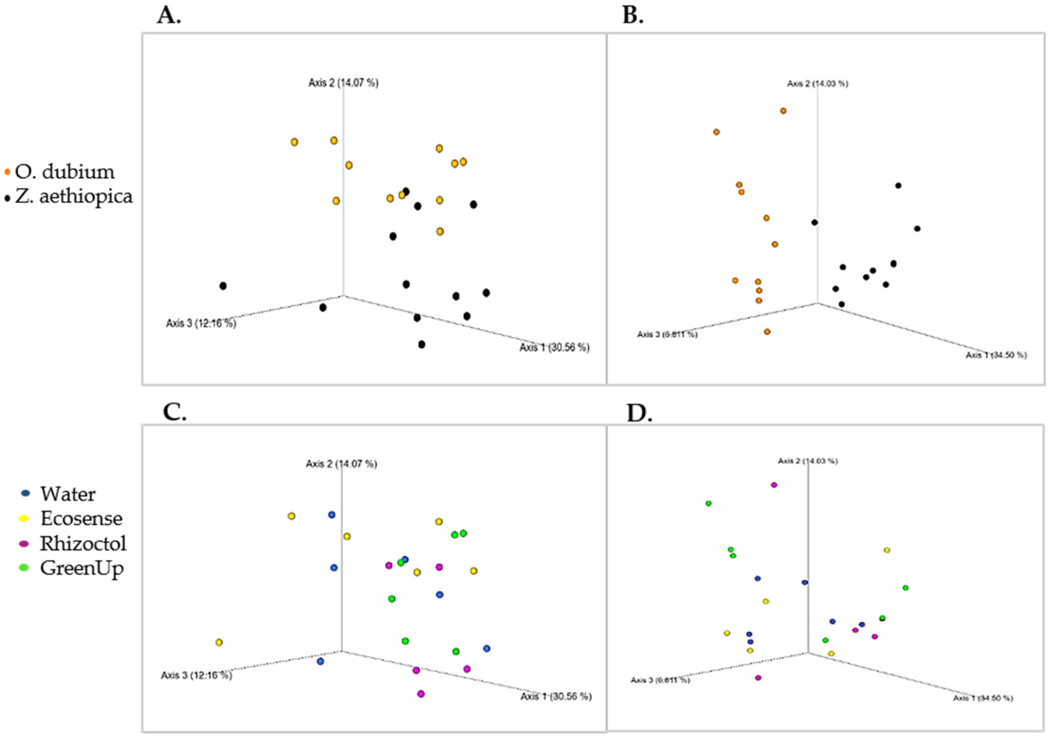

Figure S3. Principal-coordinate analysis (PCoA) plots of weighted Unifrac distance metrics of bacterial populations surrounding the roots of *Ornithogalum dubium* and *Zantedeschia aethiopica*. Data from different years were analyzed separately. (**A**) Plant species in the perlite: *O. dubium* in orange, *Z. aethiopica* in black. (**B**) Plant species in the planting mix medium: *O. dubium* in orange, *Z. aethiopica* in black. (**C**) Growth-promoting treatments in the perlite: water (control; blue), Ecosense (yellow), Rhizoctol (purple) and GreenUp (green). (**D**) Growth-promoting treatments in the planting mix: water (control; blue), Ecosense (yellow), Rhizoctol (purple) and GreenUp (green). Each data point represents sequences from a single soil sample; each plot includes all of the samples collected during that growing season.
